# MicroRNA-455-3p as a Potential Biomarker for Alzheimer's Disease: An Update

**DOI:** 10.3389/fnagi.2018.00041

**Published:** 2018-02-23

**Authors:** Subodh Kumar, P. Hemachandra Reddy

**Affiliations:** ^1^Garrison Institute on Aging, Texas Tech University Health Sciences Center, Lubbock, TX, United States; ^2^Department of Cell Biology and Biochemistry, Texas Tech University Health Sciences Center, Lubbock, TX, United States; ^3^Department of Pharmacology and Neuroscience, Texas Tech University Health Sciences Center, Lubbock, TX, United States; ^4^Department of Neurology, Texas Tech University Health Sciences Center, Lubbock, TX, United States; ^5^Speech, Language and Hearing Sciences, Texas Tech University Health Sciences Center, Lubbock, TX, United States; ^6^Department of Public Health, Graduate School of Biomedical Studies, Texas Tech University Health Sciences Center, Lubbock, TX, United States; ^7^Garrison Institute on Aging, Texas Tech University Health Sciences Center, Lubbock, TX, United States

**Keywords:** microRNA-455-3p, Alzheimer's disease, biomarker, post-mortem brains, fibroblasts, B-lymphocytes

## Abstract

A non-invasive and early-detectable peripheral biomarker is urgently needed for Alzheimer's disease (AD). The present study is a step forward to verify the biomarker properties of human microRNA-455-3p (Hsa-miR-455-3p) in AD patients. Our previous findings on mild cognitive impaired subjects, AD patients and AD cells and mouse models unveiled the miR-455-3p as a potential peripheral biomarker for AD. In the current study, we verified the differential expression of miR-455-3p in postmortem AD brains obtained from NIH NeuroBioBank, and fibroblasts and B-lymphocytes from both familial and sporadic AD patients from Coriell Cell Repository of National Institutes on Aging. Total RNA was extracted from the fibroblasts, B-lymphocytes and AD postmortem brains, and expression of miR-455-3p was measured by real-time reverse-transcriptase RT-PCR. Our real-time RT-PCR analysis showed a significant (*P* = 0.0002) upregulation of miR-455-3p expression in AD postmortem brains compared to healthy control samples. Expression of miR-455-3p was also upregulated in the fibroblasts from AD patients, however a significant difference in miR-455-3p level was observed in the cells from sporadic AD patients (*P* = 0.014) compared to healthy controls. Similarly, in B-lymphocytes, miR-455-3p level was also higher (*P* = 0.044) especially in sporadic AD cases compared to controls. Receiver operating characteristic (ROC) curve analysis indicated the significant area under ROC curve (AUROC) value of miR-455-3p in AD postmortem brain (AUROC = 0.792; *P* = 0.001) and AD fibroblasts cells (AUROC = 0.861; *P* = 0.03), whereas in B-lymphocytes AUROC value of miR-455-3p was not significant. Further, *in-silico* analysis for miRNA targets predictions showed the binding capacity of miR-455-3p with several AD associated key genes such as APP, NGF, USP25, PDRG1, SMAD4, UBQLN1, SMAD2, TP73, VAMP2, HSPBAP1, and NRXN1. Hence, these observations further revealed that miR-455-3p is a potential biomarker for AD and its possible therapeutic target for AD.

## Introduction

Alzheimer's disease (AD) is a progressive, heterogeneous, age-dependent, neurodegenerative disorder, characterized by the loss of memory, impairment of multiple cognitive functions, and changes in the personality and behavior (Mattson, 2004; LaFerla et al., 2007; Reddy et al., 2010). Currently, 36 million people older than 65 years are living with AD-related dementia world-wide, with numbers in this age group expecting to double to 66 million by 2030 and increase to 115 million by 2050. According to 2015 estimates from the World Alzheimer Report, worldwide dementia is currently costing $818 billion annually (World Alzheimer Report, 2015).

Morphological and pathological studies of postmortem AD brains revealed that AD is mainly associated with intracellular neurofibrillary tangles (NFTs); extracellular amyloid-β (Aβ) plaques; synaptic damage, loss of synapses, and loss of synaptic proteins; proliferation of reactive astrocytes and activated microglia; defects in cholinergic neurons; an age-dependent imbalance in hormones, and structural and functional changes in mitochondria (Terry et al., 1991; McGeer and McGeer, 1995; DeKosky et al., 1996; Nunomura et al., 2001; Reddy, 2006; Reddy and Beal, 2008; Du et al., 2010; Tampellini and Gouras, 2010; Swerdlow, 2011; Reddy et al., 2012; Zhu et al., 2013). Among these changes, synaptic damage and the loss of synapses and mitochondrial oxidative damage are widely recognized as early events in the pathogenesis and progression of AD (Du et al., 2010; Tampellini and Gouras, 2010). Also, the loss of synapses and synaptic damage are the best correlates of cognitive decline found in AD patients (Terry et al., 1991; Bertoni-Freddari et al., 1996; DeKosky et al., 1996).

AD occurs in two forms, early-onset “familial” AD involves genetic mutations and late-onset “sporadic” AD. Genetic mutations in amyloid precursor protein (APP), presenilin 1 (PS1), and presenilin 2 cause a small proportion of familial AD. Several factors, including lifestyle, diet, environmental exposure, apolipoprotein allele E4, and several other genetic variants reported to involve in late-onset AD (Reddy et al., 2012). Aging is a major risk factor for developing AD. With increasing lifespan, AD is becoming a major factor in the society. Currently, we do not have drugs and agents that can delay and/or prevent disease progression in elderly individuals and patients with AD. Further, we do not have peripheral biomarkers that can detect disease process early on life. However, recent advancements in molecular biology revealed that blood-based microRNAs can be used as early detectable peripheral biomarkers for aging, AD and other neurological diseases (Kumar and Reddy, 2016; Reddy et al., 2017).

MicroRNAs (miRNAs) are non-coding RNAs (20–24 nt), involved in post-transcriptional processing of genes by affecting the stability and translation of mRNAs. These are transcribed by RNA pol II in the form of capped and polyA primary transcripts (pri-miRNAs) and can be either protein-coding or non-coding. Mature miRNA sequences are incorporated into a RNA-induced silencing complex, which recognizes target mRNAs resulting in the translational inhibition or target mRNA destabilization (Kumar and Reddy, 2016). There are several miRNAs that have been implicated in most of the neurodegenerative diseases (Reddy, 2006; Williams et al., 2016).

MicroRNA-455 (MiR-455) is identified as a member of broadly conserved family non-coding RNA and expressed in most of the phylum such as Eukaryota, Metazoa, Chordata, Craniata, Vertebrata, Euteleostomi; Mammalia, and Primates etc. (www.ncbi.nlm.nih.gov). MiR-455 precursor sequence is 96 base pairs and it is present on the human chromosome 9 at locus 9q32. It is originally encoded by human COL27A1 gene (collagen type XXVII alpha 1 chain) at position 114,209,434–114,209,529. MiR-455 has been implicated in various human diseases specially cancer and chondrogenesis (Chen et al., 2016). Role of miR-455 has been identified in Human Colon Cancer (Zheng et al., 2016), prostate cancer (Zhao et al., 2017), hepatocellular carcinoma (Qin et al., 2016), gastric cancer (Liu et al., 2016), oral squamous cancer cells (Cheng et al., 2016), and non-small cell lung cancer (Li et al., 2016). However, in neurodegenerative diseases, role of miR-455-3p is barely investigated. Our previous study reported the higher expression of miR-455-3p in patients with AD and other AD sources. Current findings is the continuation of our past study to elaborate the biomarker potential of miR-455-3p in more details.

The goal of present study was to identify a suitable, non-invasive, blood-based early biomarker for AD detection. To achieve this goal, we focused on circulatory microRNAs (cmiRNAs), which are quite stable in peripheral circulation and levels of particular miRNA seems to be changing with disease severity. Our previous research findings on human serum samples from AD patients, MCI individual and healthy subjects identified significant number of deregulated miRNAs in patients compared to controls (Kumar et al., 2017). A few of them were significantly upregulated and some were down regulated in AD and MCI individual compared to healthy controls. One of the most suitable identified candidate in our study was microRNA-455-3p. Expression of miR-455-3p was found to be significantly upregulated in AD serum samples, AD postmortem brains, AD mouse model, and AD cell lines (Kumar et al., 2017). Upregulation of miR-455-3p in different cell and mouse models of AD proven its biomarker potential for AD. To further strengthen our findings, the present study is focused on the AD postmortem brains obtained from NIH NeuroBioBanks, human fibroblast, and B-lymphocytes cell lines derived from familial AD and sporadic AD patients. Expression of miR-455-3p was quantified and its diagnostic potential was examined in different sources. Further, *in-silico* analysis was performed to understand the roles and downstream application of miR-455-3p in AD. Findings from this study, will provide the valuable information about miR-455-3p role in AD and in search of pre-clinical biomarker for early AD detection.

## Materials and methods

### Study subjects

(a) AD postmortem brains—Postmortem brains from AD patients and healthy controls were obtained from three NIH NeuroBioBanks- (1) Human Brain and Spinal Fluid Resource Center, 11301 Wilshire Blvd (127A), Los Angeles, CA. (2) Brain Endowment Bank, University of Miami, Millar School of Medicine, 1951, NW 7th Avenue Suite 240, Miami, FL. (3) Mount Sinai NIH Brain and Tissue Repository, 130 West Kingsbridge Road Bronx, NY. Brain tissues were dissected from the Brodmann's Area 10 of the frontal cortices from AD patients (*n* = 27) and age and sex matched healthy controls (*n* = 15). Demographic and clinical details of study specimens were given in Table 1.

**Table 1:** Demographic and clinical details of the brain samples.

**S. No**	**Sample ID**	**Age**	**Sex**	**Neuro pathology**	**Structure**	**Autolysis time**
1	4130	67	F	Control	Broadmann's Area 10	11.8
2	4431	68	F	Control	Broadmann's Area 10	23.7
3	4660	73	F	Control	Broadmann's Area 10	18.5
4	5072	83	M	Control	Broadmann's Area 10	19.5
5	5190	68	M	Control	Broadmann's Area 10	20.3
6	HCT15HAO1713	70	M	Control	Broadmann's Area 10	12.7
7	HCTZZC1711	82	F	Control	Broadmann's Area 10	14.2
8	HCT15HBC1709	83	M	Control	Broadmann's Area 10	25
9	HCTZZT1702	84	M	Control	Broadmann's Area 10	15.5
10	HCT15HBU1704	91	F	Control	Broadmann's Area 10	18.7
11	77428	65	M	Control	Broadmann's Area 10	3.8
12	77431	103	F	Control	Broadmann's Area 10	3.8
13	77433	75	M	Control	Broadmann's Area 10	5
14	77436	93	M	Control	Broadmann's Area 10	4.1
15	77437	84	F	Control	Broadmann's Area 10	5.4
16	4513	74	M	AD	Broadmann's Area 10	15.6
17	4498	76	M	AD	Broadmann's Area 10	12.9
18	4204	68	M	AD	Broadmann's Area 10	11.9
19	4203	72	F	AD	Broadmann's Area 10	20.3
20	4454	82	F	AD	Broadmann's Area 10	9
21	4043	80	F	AD	Broadmann's Area 10	13
22	4382	74	F	AD	Broadmann's Area 10	16.2
23	4617	73	F	AD	Broadmann's Area 10	18.9
24	4718	93	F	AD	Broadmann's Area 10	8.2
25	4608	80	M	AD	Broadmann's Area 10	3.1
26	4752	89	M	AD	Broadmann's Area 10	9
27	4788	65	M	AD	Broadmann's Area 10	7.8
28	HBFR1703	69	F	AD	Broadmann's Area 10	22
29	HBFQ1711	77	M	AD	Broadmann's Area 10	18
30	HBJG1710	79	M	AD	Broadmann's Area 10	23.8
31	HBDA1704	80	M	AD	Broadmann's Area 10	22.1
32	HCTYN1713	80	F	AD	Broadmann's Area 10	6.5
33	HBDI1710	85	F	AD	Broadmann's Area 10	8
34	HBEM1701	86	M	AD	Broadmann's Area 10	15.5
35	HBIP1701	90	F	AD	Broadmann's Area 10	22.1
36	HBCG1703	90	F	AD	Broadmann's Area 10	8.5
37	HCTZX1702	95	M	AD	Broadmann's Area 10	19.8
38	77423	79	F	AD	Broadmann's Area 10	6.5
39	77424	69	M	AD	Broadmann's Area 10	5.4
40	77425	75	M	AD	Broadmann's Area 10	8
41	77426	94	F	AD	Broadmann's Area 10	4.3
42	77427	82	M	AD	Broadmann's Area 10	20.6

(b) AD patients cell lines- Human skin fibroblast and Lymphoblast cell culture systems were used for these studies. Banked skin fibroblasts and lymphoblast cells with the diagnoses AD, non-AD dementia (e.g., Huntington's disease and Parkinson's disease, and schizophrenia), and age-matched control were obtained from the Coriell Institute of Medical Research, Camden, New Jersey, USA. The demographic details of cell lines along with their passage numbers, biopsy sources and tissue types were provided in Table 2. Cells were cultured and maintained in RPMI1640 for B-lymphocytes and MEM media for Fibroblasts (Life Technologies Corporation, NY, USA; supplemented with 10% Fetal Bovine Serum and 1X penicillin/streptomycin) at 37°C with 5% CO_2_ to the 90–100% confluence stage in 25 and 75 cm^2^ cell culture flasks.

**Table 2:** Details of human Fibroblasts and B-lymphocytes.

**S. No**.	**Catalog no**	**Passage no**	**Sex**	**Age (Years)**	**Biopsy sources**	**Tissue type**	**Race**	**Disease status**
**(A) FIBROBLASTS**								
1	AG02261	11	M	61	Abdomen	Skin	Caucasian	Healthy control
2	AG16104	6	F	55	Arm	Skin	Black	Healthy control
3	AG16086	6	F	67	Arm	Skin	Other	Healthy control
4	AG12207	13	M	68	Arm	Skin	NA	Healthy control
5	AG02258	6	F	46	Lung	Lung	Caucasian	Healthy control
6	AG02262	4	M	61	Lung	Lung	Caucasian	Healthy control
7	AG06561	5	F	16FW^#^	Sacrum	Skin	Caucasian	Healthy control
8	AG12211	11	M	54	Lung	Lung	Caucasian	Healthy control
9	AG05810	11	F	79	Arm	Skin	Caucasian	Familial AD
10	AG06844	12	M	59	Arm	Skin	Caucasian	Familial AD
11	AG07613	16	M	66	Arm	Skin	Caucasian	Familial AD
12	AG09908	14	F	81	Arm	Skin	Caucasian	Familial AD
13	AG04400	19	F	61	Skin	Skin	Caucasian	Sporadic AD
14	AG06263	11	F	67	Arm	Skin	Caucasian	Sporadic AD
15	AG06264	7	F	62	Arm	Skin	NA	Sporadic AD
16	AG07375	6	M	71	Arm	Skin	Caucasian	Sporadic AD
17	AG08243	7	M	72	Arm	Skin	Caucasian	Sporadic AD
18	AG11368	15	M	77	Skin	Skin	Caucasian	Sporadic AD
**(B) B-LYMPHOCYTES**								
1	AG16639	na	M	77	Peripheral vein	Blood	Caucasian	Healthy control
2	AG11684	na	M	82	Peripheral vein	Blood	Caucasian	Healthy control
3	AG12034	na	F	80	Peripheral vein	Blood	Caucasian	Healthy control
4	AG11716	na	M	98	Peripheral vein	Blood	Caucasian	Healthy control
5	AG12032	na	M	84	Peripheral vein	Blood	Caucasian	Healthy control
6	AG16804	na	F	90	Peripheral vein	Blood	Caucasian	Healthy control
7	AG16927	na	M	85	Peripheral vein	Blood	Caucasian	Healthy control
8	AG16973	na	F	80	Peripheral vein	Blood	Caucasian	Healthy control
9	AG10673	na	F	85	Peripheral vein	Blood	Black	Healthy control
10	AG16907	na	F	88	Peripheral vein	Blood	Caucasian	Healthy control
11	AG08242	na	M	72	Peripheral vein	Blood	Caucasian	Familial AD
12	AG09905	na	M	72	Peripheral vein	Blood	Caucasian	Familial AD
13	AG09907	na	F	71	Peripheral vein	Blood	Caucasian	Familial AD
14	AG11755	na	F	85	Peripheral vein	Blood	Caucasian	Familial AD
15	AG11757	na	F	81	Peripheral vein	Blood	Caucasian	Familial AD
16	AG11758	na	M	83	Peripheral vein	Blood	Caucasian	Familial AD
17	AG06204	na	M	67	Peripheral vein	Blood	Caucasian	Sporadic AD
18	AG06868	na	F	60	Peripheral vein	Blood	Caucasian	Sporadic AD
19	AG11366	na	M	52	Peripheral vein	Blood	Caucasian	Sporadic AD
20	AG17512	na	M	70	Peripheral vein	Blood	African American	Sporadic AD
21	AG17529	na	F	86	Peripheral vein	Blood	African American	Sporadic AD
22	AG17574	na	F	83	Peripheral vein	Blood	African American	Sporadic AD

na, not available;

# *Indicates fetal week*.

### Ethical approval and consent

The study was conducted at the Garrison Institute on Aging (GIA), Texas Tech University Health Sciences Center (TTUHSC), and study protocol was approved by the Institutional Review Board of TTUHSC, Lubbock, Texas for the use of biospecimens in Project FRONTIER (IRB #: L06-028). Regarding postmortem brains and cell lines used in the current study—each of the NIH NeuroBioBanks mentioned above operated under their institution's IRB approval. As determined by the FDA Research Involving Human Subjects Committee, current study did not reach the definition of “Human Subject Research” at 45 CFR 46.102(f) and thus, 45 CFR Part 46 does not apply (Ferguson et al., 2017). Further, according to Office for Human Research Protections Guidelines biospecimens obtained by the researchers from NIGMS Human Genetic Cell Repository are not considered to be human subjects because conducting research with the samples does not involve an intervention or interaction with the individual and the samples do not contain identifiable private information (www.coriell.org).

### RNA extraction

Total RNA was isolated from the 80 mg of frontal cortices using the TriZol RT reagent (Ambion, USA) as per manufacturer instructions. Briefly, tissue samples were homogenized in 1 ml of TriZol reagent with Bio-Gen PRO200 Homogenizer (PRO Scientific Inc., CT, USA) in a 2-ml RNase-free tube. Chloroform (0.2 ml) was added to the tissue homogenate, vigorously shaken for 15 s, and stored for 5 min at room temperature. The mixture was then centrifuged at 12,000 g for 15 min at 4°C. The supernatant was transferred to a new tube and precipitated with 0.5 ml of isopropanol for 15 min at room temperature. Samples were centrifuged at 12,000 g for 10 min at 4°C. The resulting RNA pellet was washed with 1 ml of 75% ethanol and centrifuged at 7,500 g for 5 min at 4°C. The RNA pellet was dried and dissolved in 50 μl of DEPC-treated water. The quality and quantity of the RNA were analyzed by NanoDrop analysis. The value of absorbance of each brain RNA sample (A260/A280) was 1.8–2.0.

### Quantification of miRNAs expression by quantitative real-time PCR

#### Quantification involved three steps

##### Polyadenylation

One microgram of total RNA was polyadenylated with an miRNA First-Strand cDNA synthesis kit (Agilent Technologies Inc., CA, USA), following manufacturer's instructions. Briefly, a polyA reaction was prepared by mixing RNA with 4.0 μl of 5X poly A polymerase buffer, 1.0 μl of rATP (10 mM), 1 μl of *E. coli* poly A polymerase, producing a final volume of 20 μl with RNase-free water. The tube with these components was incubated at 37°C for 30 min, followed another incubation at 95°C for 5 min to terminate the adenylation reaction (Kumar et al., 2014).

##### cDNA synthesis

Ten microliters of polyadenylated miRNAs were processed for cDNA synthesis with the miRNA First-Strand cDNA synthesis kit (Agilent Technologies Inc.). The following reaction components were combined in a tube: 2 μl of 10X AffinityScript RT buffer, 0.8 μl of dNTP mix (100 mM), 1 μl of RT adaptor primer (10 μM), 1.0 μl of AffinityScript RT/RNase Block enzyme, and polyadenylated RNA. The combination resulted in a reaction volume of 20 μl RNase-free water. This reaction mixture was incubated at 55°C for 5 min, then at 25°C for 15 min, followed by an incubation at 42°C for 30 min, and a final incubation at 95°C for 5 min in a Veriti 96 well thermal cycler (Applied Biosystems, USA). Resulting cDNAs were diluted with 20 μl of RNase-free water and stored at 80°C for further analysis.

##### Real-time RT-PCR

Real-time RT-PCR reaction was performed by preparing a reaction mixture containing 1 μl of miRNA-specific forward primer (10 μm), 1 μl of a universal reverse primer (3.125 μm) (Agilent Technologies Inc., CA, USA), 10 μl of 2X SYBR® Green PCR master mix (Applied Biosystems, NY, USA), and 1 μl of cDNA. To this mixture RNase-free water was added up to a 20 μl final volume. Primers for hsa-miR-455-3p (Forward: 5′-GCAGTCCATGGGCATATACAC-3′), and U6snRNA (P1: 5′-CGCTTCGGCAGCACATATACTAA-3′ and Reverse: 5′-TATGGAACGCTTCACGAATTTGC-3′) were synthesized commercially (Integrated DNA Technologies, Inc. Iowa USA).

To normalize the miRNA expression, U6 snRNA (small nuclear RNA) expression was also quantified in the tissue and cells, which was used as an internal control. The reaction mixture of each sample was prepared in triplicates. The reaction was set in the 7900HT Fast Real Time PCR System (Applied Biosystems, USA) using following reaction conditions: initial denaturation at 95°C for 5 min, denaturation at 95°C for 10 s, annealing at 60°C for 15 s, and extension at 72°C for 25 s. The relative levels of miR-455-3p in the AD patients vs. the controls subjects were determined in terms of their fold change, using the formula (2^−ΔΔCt^), where ΔCt was calculated by subtracting Ct of U6snRNA from the Ct of miR-455-3p. Real-time RT-PCR was performed in triplicate, and the data were expressed as the mean ± *SD* (Kumar et al., 2014; Hamam et al., 2016).

### *In-silico* analysis for miR-455-3p

MiR-455-3p target genes were analyzed using various on-line miRNA algorithms (diana-microt, microrna.org, mirdb, rna22-has, targetminer, and targetscan-vert). Details about predictive and validated transcripts were obtained by searching hsa-miR-455-3p.1 and hsa-miR-455-3p.2 isoforms. Target genes were checked for following parameters: (i) their representative transcripts, (ii) number of 3P-seq tags supporting UTR +5, (iii) link to sites in UTRs, (iv) conserved sites/poorly conserved sites, (v) cumulative weighted context++ score, (vi) total context++ score, and (vii) aggregate PCT (preferentially conserved targeting) values. Further, Predicted consequential pairing showed the miRNA-target complementarity at inside or outside the seed regions of miRNAs was checked at untranslated regions links (http://www.targetscan.org).

### Statistical analysis

The real-time RT-PCR data was analyzed by using the formula 2^−ΔΔCT^ value of genes in each sample from AD patient's samples and controls. Statistical analysis was performed with Prism software, v, 6 (La Zolla, CA). *P-*value was calculated, based on the unpaired *t-*tests for analyzing two groups and using one-way comparative analysis of variance (ANOVA) when comparing between more than two groups. ROC curve was plotted based on the ΔCT value of samples in patients and control groups. Correlation analysis was performed using two tailed Pearson correlation coefficient (r) calculation considering 95% confidence interval. *P* < 0.05 was considered statistically significant.

## Results

### Up regulation of miR-455-3p expression in AD

#### AD postmortem brains

**Figure 1:**
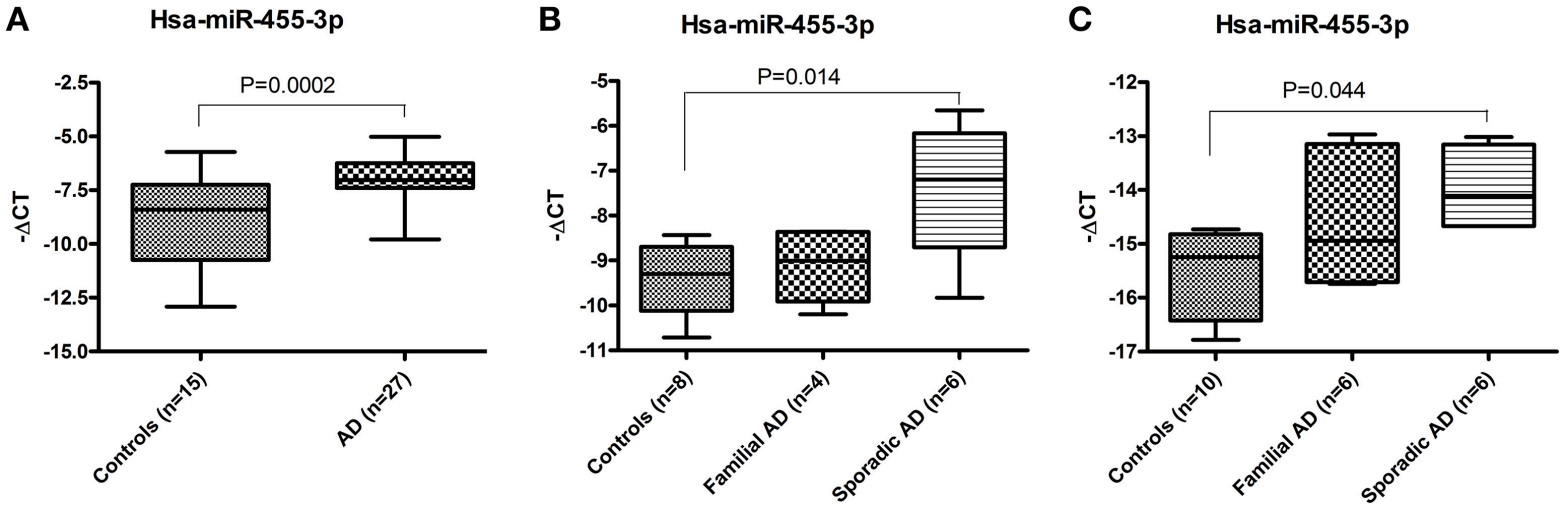
Expression of hsa-miR-455-3p in AD patients. **(A)** miR-455-3p expression in the postmortem brains of healthy controls (*n* = 15) and AD patients' (*n* = 32) was quantified by real-time RT-PCR. Data are presented as “-delta CT” values using box and whiskers plots. Significant difference between groups were calculated by unpaired *t*-test with *P* < 0.05 is considered statistically significant. **(B)** Expression of hsa-miR-455-3p in human fibroblast cells from healthy controls (*n* = 8), Familial AD cases (*n* = 4) and sporadic AD patients' (*n* = 6). Significant difference between groups were calculated by one-way ANOVA with *P* < 0.05 is considered statistically significant. **(C)** Expression of hsa-miR-455-3p in human B-lymphocytes cells from healthy controls (*n* = 10), Familial AD cases (*n* = 6) and sporadic AD patients' (*n* = 6). Significant difference between groups were calculated by one-way ANOVA with *P* < 0.05 is considered statistically significant.

Total RNA was extracted from the postmortem brains of healthy controls (*n* = 15) and AD patients (*n* = 27) and expression of hsa-miR-455-3p was quantified by real-time RT-PCR analysis. Fold-change was calculated based on the ΔCT value of miR-455-3p in AD patients' vs. healthy controls. The (–ΔCT) value (mean ± *SD*) was significantly (*P* = 0.0001) higher in AD patients (−6.89 ± 0.21) compared to the healthy controls (−8.94 ± 0.56; Figure 1A). Interestingly, fold-change analysis indicated the significantly higher expression of miR-455-3p in AD patients.

#### AD fibroblasts

Similarly, expression of miR-455-3p was quantified in the skin fibroblast cells generated form familial AD patients (*n* = 4), sporadic AD patients (*n* = 6), and healthy control subjects (*n* = 8). Differences in ΔCT values was evaluated among three groups using one-way ANOVA. Results showed the higher (−ΔCT) values (mean ± *SD*) of miR-455-3p in familial and sporadic patients compared to controls. However, significant difference (*P* = 0.014) in (−ΔCT) value was observed in sporadic cases (−7.35 ± 1.39) compared to control samples (−9.37 ± 0.76; Figure 1B).

#### AD B-lymphocytes

Further, we checked the level of miR-455-3p in B-lymphocytes obtained from familial AD patients (*n* = 6), sporadic AD patients (*n* = 6), and healthy controls (*n* = 10). The (–ΔCT) (mean ± *SD*) value was compared among three group using one-way ANOVA. Analysis showed the variations in miR-455-3p level among these groups, however significant difference (*P* = 0.044) in (–ΔCT) value was reported between sporadic AD cases (−13.98 ± 0.73) and controls (−15.50 ± 0.80; Figure 1C). Hence, results obtained from AD postmortem brains, AD fibroblast, and AD B-lymphocyte were conclusively confirmed the decisive role of miR-455-3p in AD assessment.

### Receiver operating characteristics curve analysis of miR-455-3p

#### AD postmortem brains

**Figure 2:**
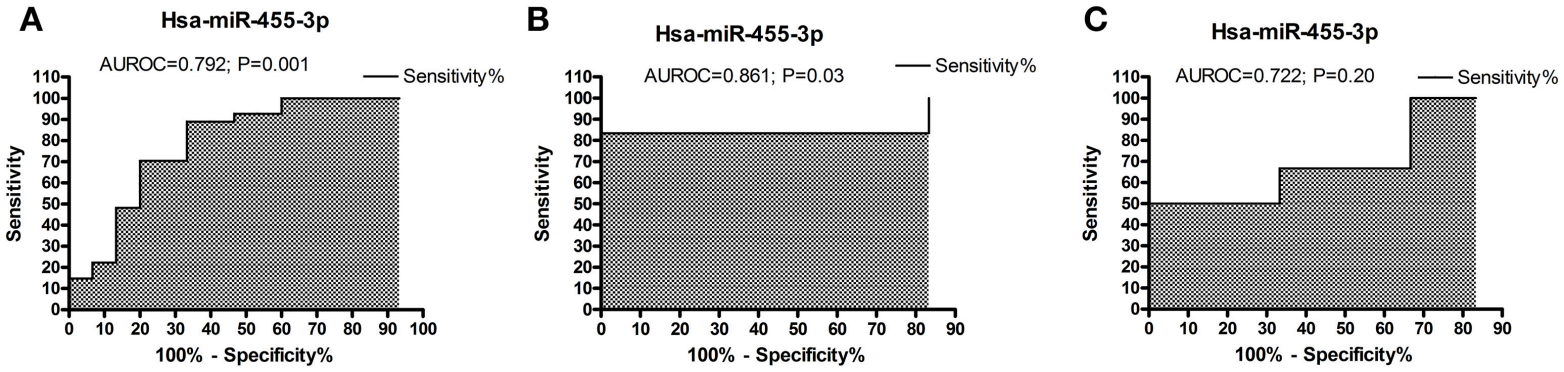
ROC curve analysis of hsa-miR-455-3p in **(A)** AD postmortem brains, **(B)** AD fibroblast cell lines, and in **(C)** B-lymphocytes cells from AD patients. The curve was plotted based on the ΔCT value of miR-455-3p in AD patients and control samples. Area under the ROC curve (AUROC) was calculated along with the sensitivity and specificity values. *P* < 0.05 is considered statistically significant.

To determine the diagnostic performance of miR-455-3p expression in AD patients, ROC curve was plotted using (ΔCT) values of miR-455-3p in AD patients and healthy controls. Analysis showed the significant area under ROC curve (AUROC) value of miR-455-3p (AUROC = 0.792) with the 95% confidence interval was 0.637–0.948 (*P* = 0.0018). The cut-off value was 8.16 with sensitivity of 88.89% (95% confidence interval: 70.84–97.65%) and specificity was 66.67% (95% confidence interval: 38.38–88.18%) in AD brain samples compared with healthy controls (Figure 2A).

#### In AD fibroblasts

ROC curve was analyzed for miR-455-3p expression in fibroblast cells form familial and sporadic AD patients vs. healthy controls. However, significant AUC value was obtained for ROC curve when comparing between sporadic AD patients with healthy controls. AUROC value was (0.861) with 95% confidence interval of 0.6036–1.119 (*P* = 0.037). The cut-off value was 9.12 with sensitivity of 83.33% (95% confidence interval: 35.88–99.58%) and specificity was also 66.67% with confidence interval (22.28–95.67%; Figure 2B).

#### In AD B-lymphocytes

Similarly ROC curve for miR-455-3p was analyzed in B-lymphocytes of AD patients. Analysis between sporadic AD patients and healthy controls showed the fair AUROC value (0.722) with 95% confidence interval of 0.4185–1.026 (*P* = 0.20). The cut-off value was 14.90 with sensitivity of 66.67% (95% confidence interval: 22.28–95.67%) and specificity was 50.00% (95% confidence interval: 11.81–88.19%; Figure 2C). Thus, analysis showed that ROC analysis of miR-455-3p in B-lymphocytes was not significant. However, data from postmortem AD brains and AD fibroblasts cells showed significant ROC curve data further confirmed that the miR-455-3p as a valuable molecule capable of discriminating the patients with AD from healthy individuals.

### Correlation of miR-455-3p expression with patients' demographic data

**Figure 3:**
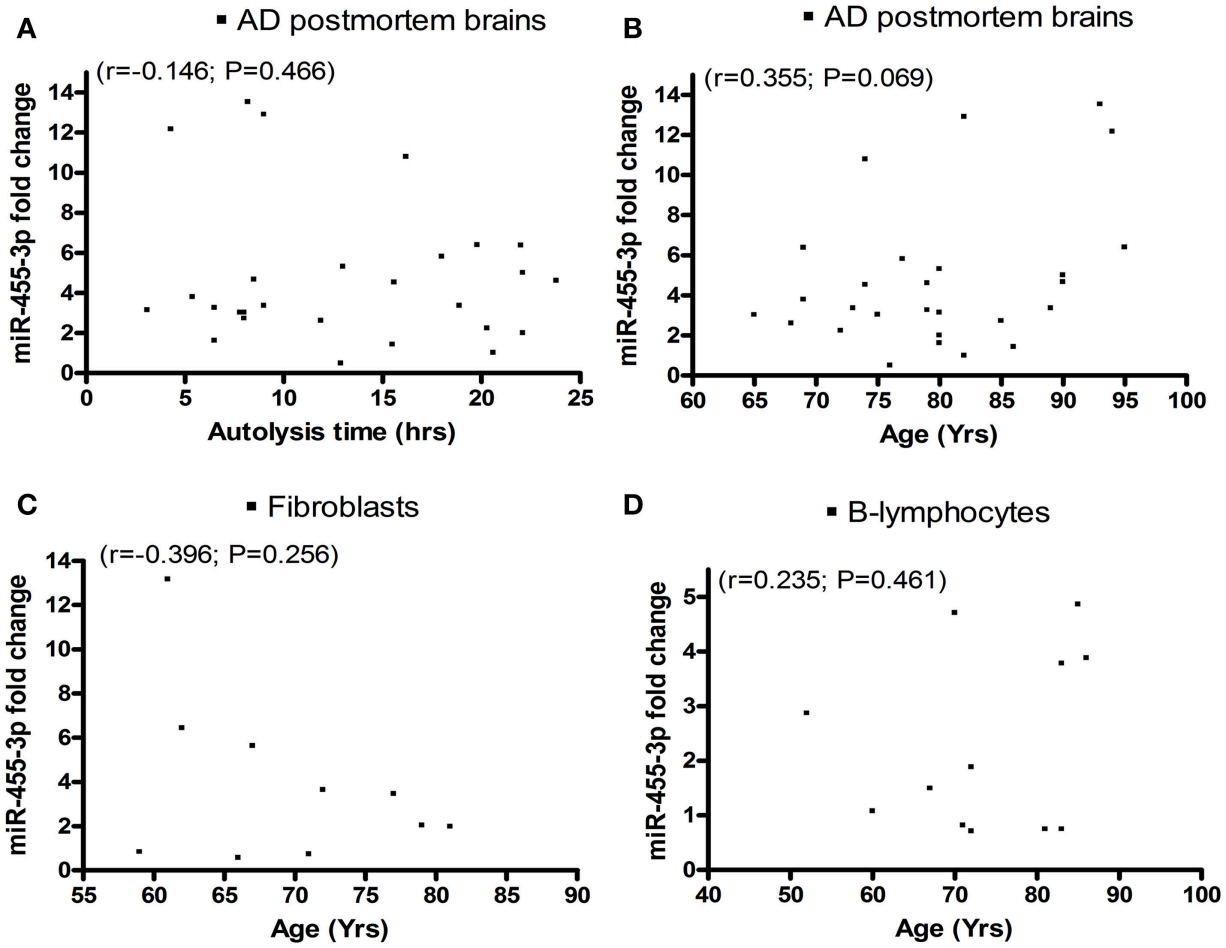
Scattered plot diagrams showing the Pearson correlation coefficient (r) values of miR-455-3p expression with **(A)** AD postmortem brains autolysis time **(B)** Age of AD postmortem brains **(C)** Age of AD fibroblast cells and **(D)** Age of AD B-lymphocytes.

We analyzed miR-455-3p expression levels in relation to (1) postmortem interval, (2) AD patients' age, and also (3) donors' age of fibroblasts, and (4) B-lymphocytes using Pearson correlation coefficients (r). AD postmortem brains showed a negative correlation *r* = −0.146 (with 95% confidence interval: −0.498 to 0.247; *P* = 0.466) between brains postmortem interval and miR-455-3p expression level (Figure 3A). Whereas a positive correlation *r* = 0.355 (with 95% confidence interval: −0.029 to 0.647; *P* = 0.069) was observed between the age of AD postmortem brains and miR-455-3p level (Figure 3B). However, *P*-values were not significant in both cases. Thus, results showed a trend of reduced levels of miR-455-3p with increased postmortem interval and increased trend of miR-455-3p with patients' age. As shown in Figures 3C,D, donors' age for fibroblasts (*r* = −0.396, 95% confidence interval: −0.821 to 0.310; *P* = 0.256), and B-lymphocytes (*r* = 0.235, 95% confidence interval: −0.391 to 0.713; *P* = 0.461), we did not find statistical significance, between donors age with miR-455-3p levels for fibroblasts and B-lymphocytes, indicating that donors age do not affect miR-455-3p expression levels.

### *In-silico* analysis for miR-455-3p function in AD

*In-silico* analysis was performed to understand the functions of miR-455-3p and its possible role in AD pathogenesis. Analysis was performed using the various bio-informatics algorithms such as DIANA-MICROT, MICRORNA.ORG, MIRDB, RNA22-HAS, TARGETMINER, and TARGETSCAN-VERT. As per miRbase database, a total of 3,102 reads of miR-455-3p has been detected by deep sequencing in 62 experiments (www.mirbase.org). Each algorithm was run for miR-455-3p and validated/predictive target genes were analyzed. A total of 323 predicted transcripts/human genes were identified with conserved miR-455-3p.2 binding site. Out of these genes most potential 13 targets genes were screened for those were having the roles in AD pathogenesis. Important ones were: APP, NGF, USP25, PDRG1, SMAD4, UBQLN1, SMAD2, TP73, VAMP2, HSPBAP1, and NRXN1 (Table 3). miR-455-3p having at least one or two binding site at 3′UTR of the genes and total context^++^ score ranges from −0.1 to −0.46. For e.g., miR-455-3p binds at the two sequence sites of 3′ UTR of APP gene at sequence position 522–528 and 3,139–3,145. Interaction of miR-455-3p at these sites will influence the expression level of APP genes. Hence, these analyses indicated the possible way the miR-455-3p involved in AD pathogenesis.

**Table 3:** Predictive/validated gene targets of miR-455-3p involved in AD.

**S. No**	**Representative miRNA**	**Ortholog of target gene**	**Representative transcript**	**Gene name**	**3P-seq tags + 5**	**Conserved sites total**	**Cumulative weighted context++ score**	**Total context++ score**	**Aggregate PCT**
1	hsa-miR-455-3p.2	NGF	ENST00000369512.2	Nerve growth factor (beta polypeptide)	27	1	−0.46	−0.46	0.38
2	hsa-miR-455-3p.2	USP25	ENST00000285681.2	Ubiquitin specific peptidase 25	2012	2	−0.45	−0.45	0.6
3	hsa-miR-455-3p.2	PDRG1	ENST00000202017.4	p53 and DNA-damage regulated 1	116	1	−0.45	−0.45	<0.1
4	hsa-miR-455-3p.2	SMAD4	ENST00000398417.2	SMAD family member 4	403	2	−0.3	−0.32	<0.1
5	hsa-miR-455-3p.2	UBQLN1	ENST00000376395.4	Ubiquilin 1	471	2	−0.3	−0.33	<0.1
6	hsa-miR-455-3p.2	APP	ENST00000346798.3	Amyloid beta (A4) precursor protein	4570	2	−0.29	−0.35	<0.1
7	hsa-miR-455-3p.1	SMAD2	ENST00000262160.6	SMAD family member 2	1196	2	−0.2	−0.28	0.33
8	hsa-miR-455-3p.1	TP73	ENST00000378280.1	Tumor protein p73	831	1	−0.14	−0.14	0.3
9	hsa-miR-455-3p.1	VAMP2	ENST00000316509.6	Vesicle-associated membrane protein 2 (synaptobrevin 2)	1840	1	−0.11	−0.11	0.26
10	hsa-miR-455-3p.1	HSPBAP1	ENST00000383659.1	HSPB (heat shock 27kDa) associated protein 1	22	1	−0.11	−0.15	<0.1
11	hsa-miR-455-3p.1	NRXN1	ENST00000342183.5	Neurexin 1	5	1	−0.1	−0.1	0.3

## Discussion

The purpose of our study was to determine the blood-based peripheral biomarkers for AD. We recently conducted a high throughput microRNA analysis using serum-derived RNA samples from MCI subjects, AD patients, and healthy control subjects (Kumar et al., 2017). We found several differentially expressed miRNAs in MCI subjects and patients with AD relative to healthy controls. Further, we verified differentially expressed miRNAs using real-time RT-PCR from serum-derived miRNAs, and also from cell and mouse models of AD. In the current study, we extended our investigations using large numbers of fibroblasts, B-lymphocytes from familial and sporadic AD patients and age-matched control subjects. We found miR-455-3p levels were upregulated in the fibroblasts and B-lymphocytes from AD patients relative to healthy control subjects. However, a significant difference was observed in the cells form sporadic AD patients compared to healthy controls. Similarly, in B-lymphocytes, miR-455-3p level was significantly upregulated in sporadic AD cases compared to controls (*P* = 0.044). Receiver operating characteristic curve analysis indicated the significant area under curve value of miR-455-3p in AD postmortem brains (AUROC = 0.792; *P* = 0.001) and AD fibroblasts cells (AUROC = 0.861; *P* = 0.03). These observations strongly suggest that miR-455-3p is a potential biomarker for sporadic AD.

**Figure 4:**
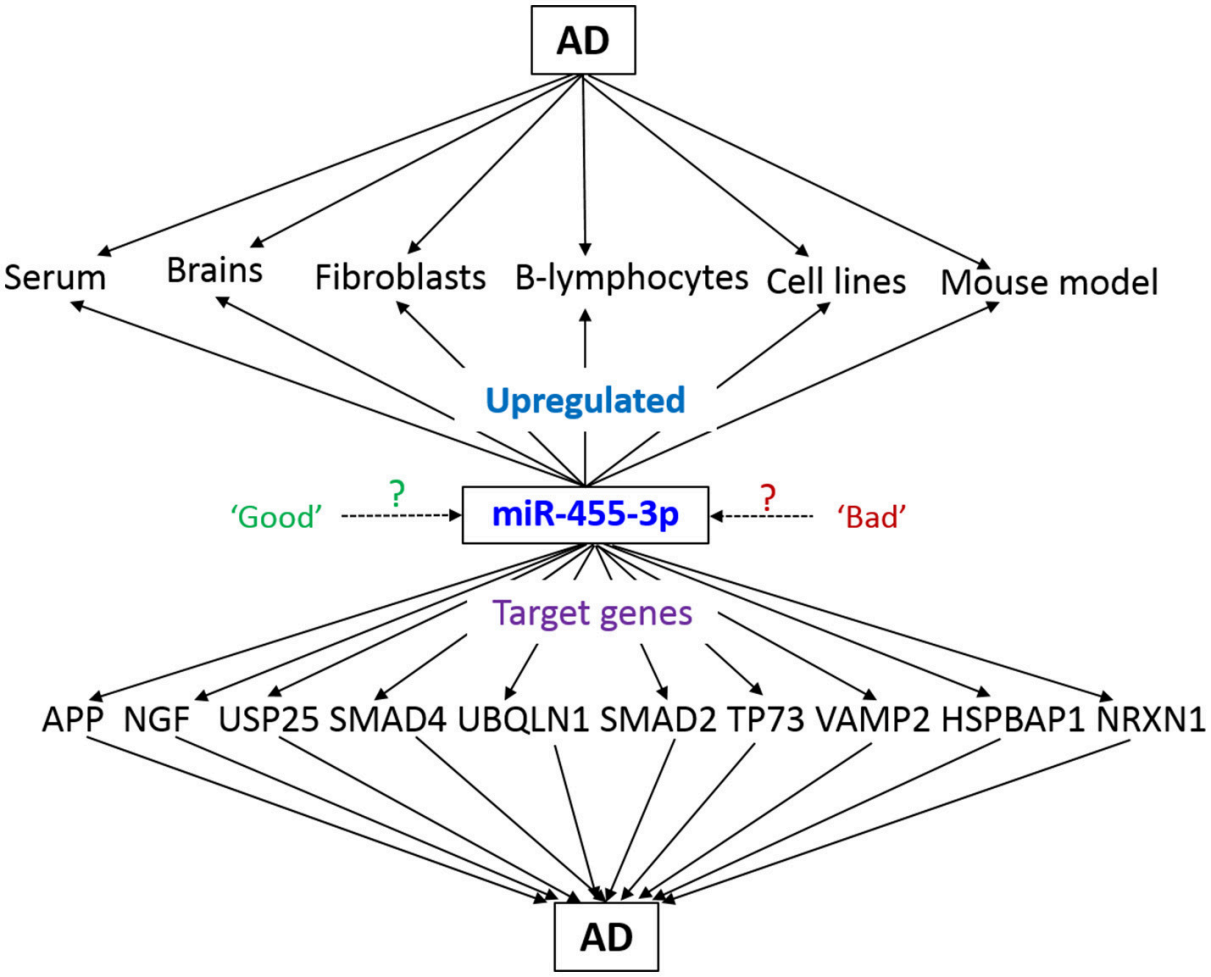
Schematic representation of miR-455-3p roles in AD pathogenesis. miR-455-3p expression is upregulated in different AD models and sources. Simultaneously, miR-455-3p target several key genes those are involved in the AD. Hence, upregulation of miR-455-3p in AD may be a “good” or “bad” signal for the cells. However, exact molecular link between miR-455-3p, AD, and target genes needs to be determined.

An early stage pre-clinical diagnostic biomarkers are urgently needed to detect disease process early on in life and take necessary action to prevent and/or delay disease progression. Recent molecular biology studies using serum/plasma revealed that several circulatory microRNAs can be used as potential peripheral biomarkers for AD (Kumar and Reddy, 2016). However, these circulatory microRNAs are needed further validation using postmortem AD brains and cell and mouse models of AD. Therefore, more accurate and mechanistic research is needed to determine potential candidates as biomarkers for AD. As mentioned above, our recent lab study on AD serum samples and other AD sources/AD mouse model unveiled the miR-455-3p as potential biomarker candidate for AD (Kumar et al., 2017; Figure 4). Many other reports identified the role of miR-455-3p in several cancers and chondrogenic differentiation (Chen et al., 2016; Cheng et al., 2016; Li et al., 2016; Liu et al., 2016; Qin et al., 2016; Zheng et al., 2016; Zhao et al., 2017). Our study was the first to reveal the higher expression level of miR-455-3p in persons with AD.

Current study is the continuation of our ongoing biomarkers research project in the Reddy Lab. Here, first we investigated miR455-3p levels in the well-defined postmortem brain tissues from AD patients. All tissues were dissected from the affected area (Broadmann's area 10) of AD patients and commonly used for the investigation of AD pathogenesis (Wilcock et al., 2015; De Rossi et al., 2016; Shackleton et al., 2017). Our current study on AD postmortem findings revealed that miR-455-3p levels are significantly increased in a large number (*n* = 27) of AD brains and a significant AUC value also strengthen its biomarker potential. However, we don't know the exact reason for the upregulation of miR-455-3p in AD brains and further, we still do not know molecular mechanism(s) of its increased levels. Based on current findings, we cautiously propose that the upregulation of miR-455-3p—may be a compensatory to the amyloid beta toxicity in disease process. However, we need further research to understand the nature of miR-455-3p upregulation, not only serum but also in affected tissues from AD patients and AD cell and mouse models.

Beside brain tissues, we also investigated the AD fibroblasts and B-lymphocytes for miR-455-3p expression. These AD cell lines are the good sources for the investigation of AD pathologies and associated molecular changes in the patients' genome (Khan and Alkon, 2016). Both cell types showed the significantly higher levels of miR-455-3p, especially in sporadic AD cases but not in familial AD. Further, high level of miR-455-3p in AD fibroblasts and lymphoblasts indicate that increased levels of miR-455-3p is a typical feature of AD—both in the brain and peripheral cells. Alteration of miR-455-3p expression in AD cell lines indicates the strong molecular association of miR-455-3p with AD progression.

In order to expose the roles and functions of miR-455-3p in AD, *in-silico* analysis provides the valuable information. As described 11 genes were reported to involve in AD progression (Figure 3) (Burton et al., 2002; Li et al., 2004; Slifer et al., 2006; Tóth et al., 2013; Sindi et al., 2014; Jung et al., 2015, 2016; Malkki, 2015; Vallortigara et al., 2016; Kuruva et al., 2017). To understand the roles of miR-455-3p in AD, expression of these genes needs to be studied by using miR-455-3p modulation approaches (mimics/inhibitors). In this direction, next phase of our study is to determine the effect of miR-455-3p on its AD related target genes. Current focus of our laboratory is to understand the role of miR-455-3p in APP processing and amyloid beta modulation, using miR-455-3p mimics and inhibitor treatments. We also predict that two potential binding sites of miR-455-3p at the 3′UTR of APP gene may be involved in the modulation of full-length APP. Further, we also predict that miR-455-3p affects the APP processing and amyloid beta production. Therefore, current findings together with our recently published study in *Human Molecular Genetics* (Kumar et al., 2017), strongly suggest that miR-455-3p as a potential biomarker and a possible therapeutic candidate for AD.

In summary, for the first time, we report that microRNA455-3p as a potential peripheral biomarker for AD. Our research findings are based on (1) blood-based circulatory microRNAs from AD patients, (2) AD postmortem brains, AD cell lines, and AD mouse models and a large number of AD fibroblasts and lymphoblasts.

## Author contributions

PR has contributed in designing the concept of the paper and contributed to write up of the paper. SK has performed the experiments and contributed to write up of the paper.

### Conflict of interest statement

The authors declare that the research was conducted in the absence of any commercial or financial relationships that could be construed as a potential conflict of interest.
